# Use of the 9-item Shared Decision Making Questionnaire (SDM-Q-9 and SDM-Q-Doc) in intervention studies—A systematic review

**DOI:** 10.1371/journal.pone.0173904

**Published:** 2017-03-30

**Authors:** Hanna Doherr, Eva Christalle, Levente Kriston, Martin Härter, Isabelle Scholl

**Affiliations:** 1 Department of Medical Psychology, University Medical Center Hamburg-Eppendorf, Hamburg, Germany; 2 The Dartmouth Institute for Health Policy and Clinical Practice, Dartmouth College, Lebanon, New Hampshire, United States of America; TNO, NETHERLANDS

## Abstract

**Background:**

The Shared Decision Making Questionnaire (SDM-Q-9 and SDM-Q-Doc) is a 9-item measure of the decisional process in medical encounters from both patients’ and physicians’ perspectives. It has good acceptance, feasibility, and reliability. This systematic review aimed to 1) evaluate the use of the SDM-Q-9 and SDM-Q-Doc in intervention studies on shared decision making (SDM) in clinical settings, 2) describe how the SDM-Q-9 and SDM-Q-Doc performed regarding sensitivity to change, and 3) assess the methodological quality of studies and study protocols that use the measure.

**Methods:**

We conducted a systematic review of studies published between 2010 and October 2015 that evaluated interventions to facilitate SDM. The search strategy comprised three databases (EMBASE, PsycINFO, and Medline), reference tracking, citation tracking, and personal knowledge. Two independent reviewers screened titles and abstracts as well as full texts of potentially relevant records. We extracted the data using a pilot tested sheet, and we assessed the methodological quality of included studies using the Quality Assessment Tools from the U.S. National Institute of Health (NIH).

**Results:**

Five completed studies and six study protocols fulfilled the inclusion criteria. The measure was used in a variety of health care settings, mainly in Europe, to evaluate several types of interventions. The reported mean sum scores ranged from 42 to 75 on a scale from 0 to 100. In four studies no significant change was detected in the mean-differences between main groups. In the fifth study the difference was small. Quality assessment revealed a high risk of bias in four of the five completed studies, while the study protocols received moderate quality ratings.

**Conclusions:**

We found a wide range of areas in which the SDM-Q-9 and SDM-Q-Doc were applied. In the future this review may help researchers decide whether the measure fits their purposes. Furthermore, the review revealed risk of bias in previous trials that used the measure, and may help future trials decrease this risk. More research on the measure’s sensitivity to change is strongly suggested.

## Introduction

Shared decision making (SDM) is promoted in many health care systems and is gaining importance internationally [1–3]. Reasons for these changes include patients’ expanding knowledge of diseases and treatments through media, increasing numbers of available treatment options, and patients’ and physicians’ preferences for more active patient involvement [4–8]. SDM involves at least one patient and one health care provider (HCP). Both parties take steps to actively participate in the process of decision making, share information and personal values, and together arrive at a treatment decision with shared responsibility.

SDM is indicated if there are multiple possible treatments and the alternatives have different and uncertain outcomes, as is the case in most chronic diseases [9–12], or if the treatment outcome is considered subjectively important [13–15]. SDM can help patients and HCPs reach treatment agreement in long-term decisions [9, 14]. Greater patient involvement in treatment decisions is associated with less decisional conflict, which can be viewed as a moderator for patient satisfaction [16]. SDM is associated with feelings of autonomy, control, and individual competence [17]. Still, more research is needed on the general effects of SDM [18]. Interventions to facilitate SDM are becoming increasingly important, and their results need to be assessed and measured.

Measurements for SDM can be categorised by decision antecedents (e.g., role preference), the decision process (e.g., observed or perceived behaviour of the clinician), or decision outcomes (e.g., decisional conflict, decisional regret, satisfaction)[16]. The SDM process can be assessed by an external observer, the patient, or the physician; a complete overview is given in a 2010 review [19]. The OPTION ("observing patient involvement") scale is the most prominent instrument for assessing the extent to which clinicians actively involve patients in decision-making [20]. Due to several shortcomings this scale was recently revised to a short form that assesses the SDM process from an observer’s perspective in just five items [21]. Furthermore, several measures exist to assess the patient’s perspective. Among the most well known are the Perceived Involvement in Care (PICS) scale [22] and the recently developed ColloboRATE measure [23]. Although SDM is conceptualized as a process involving both the health care provider and the patient, only a few scales are available that assess SDM from both the patient’s and the physician’s points of view: the dyadic OPTION scale [24], the MAPPIN’SDM measure [25] and the 9-item Shared Decision Making Questionnaire (SDM-Q-9), published in 2010 [11]. Of the three measures, the SDM-Q-9 is used increasingly often to assess interventions aiming to improve SDM. This is likely due to its psychometric testing, acceptance, and feasibility of administration with only nine items [19]. The SDM-Q-9 is a patient-reported measure that focuses on the decisional process by rating physicians’ and patients’ behaviour in medical encounters. It was developed as a revision of the original Shared Decision Making Questionnaire (2006) [11]. The research team (including several of the authors of this manuscript, i.e. LK, MH, and IS) [11] generated a new core set of items based on the model by Elwyn et al. (2000) [26], from which nine items were selected via statistical analysis. The measured construct was found to be largely unidimensional. The answering scale was adjusted from 4-point to 6-point ratings with extremes (“completely disagree” to “completely agree”) to counter high ceiling effects [11]. The SDM-Q-9 showed good internal consistency (α = .94) and high face and structural validity in its first psychometric testing in a large (N = 2,351) primary care sample [11]. The same core research team created the physician version of the SDM-Q-9, the SDM-Q-Doc, which measures the same aspects of SDM, but from the physician’s perspective [27]. They maintained similar wording and used the same 6-point Likert scale as response format. Psychometric testing showed a high level of acceptance, with 93% completion rate for all items. The item-difficulty ranged from 3.52 to 4.34 on a scale from 0 to 5. The scale showed a good internal consistency (α = .88) and a good model-fit in a confirmatory factor analysis. [27]. With the quick and easy to answer SDM-Q-9 and SDM-Q-Doc, a dyadic (bi-perspective) measurement of SDM became possible [27].

The SDM-Q-9 was translated into English [11, 27], allowing for use in international research. The English version was tested in a stratified primary care sample (N = 488) in the U.S and confirmed a unidimensional structure and high internal consistency [19]. Further psychometric testing of the English version in a representative sample of the US population (N = 1,341) revealed discriminative validity of the SDM-Q-9, which had not been tested before [23]. A range of further translations have been conducted (see www.sdmq9.org). Several of the translations have undergone psychometric testing. In a Dutch psychometric study, both the SDM-Q-9 (sample of N = 182 outpatients) and the SDM-Q-Doc (sample of 43 primary care physicians and specialists rating N = 201 consultations) showed good reliability and convergent validity [28]. Factor analysis showed difficulties with integrating item 1 (“My doctor made clear that a decision needs to be made”) into the one-component model found by the original authors [28]. Psychometric testing of the Spanish version [29] in a sample of primary care patients with chronic conditions (N = 540) also yielded good reliability, while indicating that the best model fit was found when excluding item 1, which is consistent with the Dutch results. Furthermore, testing of the Persian version of the SDM-Q-Doc showed good reliability in a sample of hospital doctors [30]. Finally, a recent psychometric testing of the Hebrew version in a sample of mental health patients (N = 101) showed good reliability, convergent validity, a one factorial structure, and sensitivity to change [31]. While results consistently show good reliability, as well as good evidence for convergent validity, results regarding the factorial structure indicate mixed findings for item 1. Furthermore, initial studies indicating discriminative validity [23] and sensitivity to change [31], need to be confirmed by further studies. The availability of the measure in multiple languages with a relatively large amount of psychometric testing broadened the possibilities of its use in different health care systems. This may allow for examination of cross-country effects in the near future. So far, no systematic review gives an overview on the use of the 9-item Shared Decision Making Questionnaire in intervention studies.

The aims of this systematic review were to 1) evaluate the use of the SDM-Q-9 and SDM-Q-Doc in intervention studies on SDM interventions in clinical settings, 2) describe how the SDM-Q-9 and SDM-Q-Doc performed regarding sensitivity to change, and 3) investigate the methodological quality of studies and study protocols using the measure.

## Methods

Before starting with the systematic review, the authors drafted a protocol for their own use. The protocol was not registered or published. The content of the protocol is equivalent to the content of the methods described in this paper. The PRISMA checklist of the review can be found in S8 Table.

### Search strategy

We performed an electronic literature search in the databases EMBASE, PsycINFO, and Medline. We included all articles published between January 2010, the year in which the 9-item Version of the Shared Decision Making Questionnaire (SDM-Q) [11] was published, and October 13th, 2015. We devised a search strategy for this primary search encompassing all possible variations of the name of the measure. The detailed lists of keywords used can be found in the S1 Appendix. Eligibility criteria are displayed in Table 1. We performed a secondary search via the Web of Knowledge and Google Scholar including citation tracking of the original articles on the SDM-Q-9 and SDM-Q-Doc [11, 27] as well as on articles on the validation of other language versions of the questionnaire [28, 29]. We performed additional reference tracking on reviews of SDM intervention studies[32–34]. Furthermore, we contacted researchers known to be working with the measure (based on requests from the developers) to ask if they had published work using either instrument. Finally, we sent an open request for studies using the SDM-Q-9 and/or SDM-Q-Doc to a social media SDM interest group.

**Table 1:** Inclusion and exclusion criteria.

Inclusion criteria	Excluded full texts (N = 69)
1 The full text is accessible	—^a^
2 The article is published in a peer-reviewed journal	7
3 The language of the publication is English or German	—
4 The publication date is between 2010 and 2015	—
5 The type of article is an original study or a study protocol	6
6 The SDM-Q-9 and/or SDM-Q-Doc is used in the study	52
7 The participants included in the study are adults	1
8 The SDM-Q-9 and/or SDM-Q-Doc was used as an outcome measure to evaluate an intervention	3
**Exclusion criterion**	
1 The single aim of the study was to test psychometric properties of SDM-Q-9 and/or SDM-Q-Doc	—

^a^— = no full text was excluded for this reason.

### Study selection

We imported all identified records into a reference management software. After removal of duplicates, HD and IS performed an independent title and abstract screening to check for potential inclusion of records. A record was included into the next step if at least one reviewer deemed it appropriate. The full texts of the potentially relevant records were assessed independently for eligibility by HD and IS. In the case of disagreement, it was planned to discuss the respective full text with a third reviewer. However, no disagreement occurred during full-text screening.

### Data extraction

Preliminary data extraction sheets were developed by HD, discussed with IS and pilot tested by HD. HD extracted information on descriptive data of the included studies and protocols, e.g. study aims, study designs, health care settings, samples, evaluated interventions, statistical analyses, results, and interpretations. For complete data extraction sheets please see S6 Table and S7 Table. The final data extraction was conducted by one reviewer (HD) for two reasons: a) pilot testing revealed that this strategy was feasible, and b) the review team faced limited resources for data extraction.

Considering the substantial clinical and methodological heterogeneity of the set of included studies, we decided that they estimated the same parameter of interest broadly rather than specifically. This implies that a meta-analytic effect estimate would likely to be prone to numerous sources of bias. We decided that under theses circumstances a narrative-qualitative summary was more appropriate than a meta-analysis [35].

### Quality assessment

Study quality was assessed using the Quality Assessment Tools from the Risk Assessment Workgroup (2013) of the U.S. Department of Health and Human Services from the U.S. National Institute of Health (NIH) [36]. These tools were constructed to assess the internal validity of a trial, the extent to which the reported effects can truly be attributed to the intervention utilized, and the potential flaws in methodology or implementation. The reviewer can select from the response options “yes”, “no”, or “cannot determine (CD)/not reported (NR)/not applicable (NA)”. Studies are judged to be of “good”, “fair” or “poor” quality. In the present review, the tools for before-after (pre-post) studies with no control group, controlled intervention studies, and observational-cohort and cross-sectional studies were used for independent quality appraisal by HD and EC. Differences in ratings were resolved by discussion until an agreement was reached.

After rating one study and one study protocol, it became apparent that the tools needed to be slightly adapted in wording for the rating of the study protocols, (e.g., from past tense to future tense). Three criteria of the assessment tool for *controlled intervention studies* were left out in the rating of study protocols, as they were inapplicable for protocols (e.g. drop-out rates). Likewise, it became evident that the tool for controlled intervention studies was not sufficient for the quality assessment of cluster randomised controlled trials (cRCTs), as it was developed for individually randomised controlled trials (RCTs). We adapted the tool for cluster randomisation by adding five items, based on literature on the methodology of cRCTs [37–43] (see S1 Table).

Additionally, since blinding for HCPs was seldom feasible in cRCTs, item 4 assessing the blinding of participants and HCPs was divided into two items: 4a) participants and 4b) HCPs. As this review focuses on the SDM-Q-9 and SDM-Q-Doc, item 5, which considers whether the researchers assessing the outcomes are blinded to the participants’ group assignments, was changed to ascertain whether the patients or HCPs filling in the SDM-Q-9 and/or SDM-Q-Doc were blinded to the treatment group assignments. Finally, we left out item 11, which was not applicable for the aims of this review. See S1 to S5 Tables for final items.

All changes were pilot tested independently by HD and EC. Differing judgments were resolved by discussion.

## Results

### Literature search and study selection

**Fig 1:**
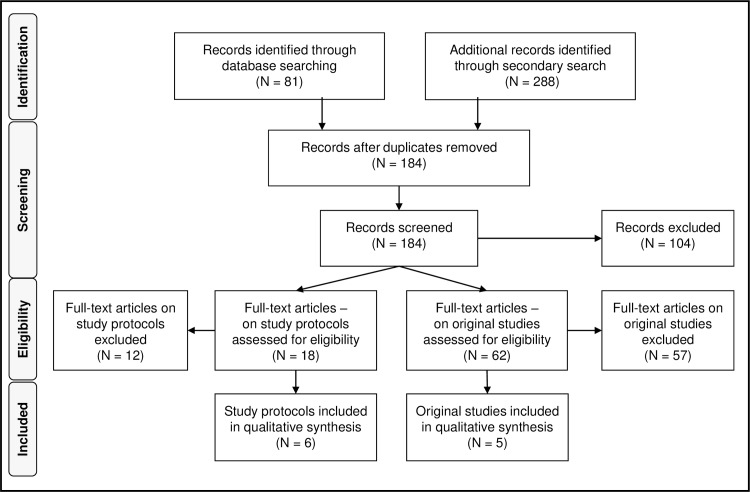
Flow diagram of study selection.

After removal of duplicates 184 records underwent title and abstract screening, which led to the exclusion of 104 records. The full texts of the remaining 80 records were assessed for eligibility. A total of 69 records were excluded after applying the inclusion and exclusion criteria (see Table 1). As a result, we included 6 study protocols and 5 original studies in this review, for a total of 11 records. As is shown in Table 1 most of the records were excluded because they did not use the SDM-Q-9 and/or SDM-Q-Doc in their study (N = 52). The overview of the procedure is given in the flow diagram, Fig 1.

### Description of included original studies

The characteristics of the original studies are displayed in Table 2 and Table 3. Three of the five included studies were cRCTs [44–46]. All but one study [47] were done in Germany. The studies were conducted in different settings and different decisional contexts. All studies had at least two measurement time points. Two of five used both measures, SDM-Q-9 and SDM-Q-Doc [45, 47] and two studies [44, 45] reported adaptation of the questionnaire for all health care professionals (HCPs). Three of five studies reported applying the measure directly after the clinician-patient-consultation [45–47]. While one study evaluated an intervention on both patients and physicians (decision aid & training) [47], four studies evaluated training programs for HCPs only. The sample sizes ranged from N = 51 patients to N = 2,188 patients, and mean ages ranged from 42.8 to 65.0 years. The highest percentage of women per group was 80% [47] and the lowest was 33% [45]. The HCP samples were described in less detail; the studies by Körner et al. reported on age and gender [44, 45]. The reported mean sum scores of the SDM-Q-9 and SDM-Q-Doc ranged from 42 to 75 on a scale from 0 to 100. Three studies did not find a significant intervention effect and concluded that the investigated interventions were ineffective [46–48]. Körner et al. 2012 found no overall intervention effect, but subgroup analyses revealed highest effects for female HCPs and for nurses [44]. Körner et al. 2014 found a small intervention effect for staff, which was highest for nurses, as well [45]. For complete data extraction sheet of original studies please see S6 Table.

**Table 2:** Characteristics of the included original studies.

**First author (year), country**	study objectives*	**study design**	**health care setting**	**SDM-Q-9 &/-Doc (assessment point)**	**Primary or secondary outcome**	**intervention**	**decisional context**
Brito et al. (2015), USA	test a DA	pre-& post- implementation study	outpatient specialty care	SDM-Q-9 &-Doc (directly after consultation)	primary	DA & training of physicians	Graves’ Disease
Hölzel et al, (2012), Gerrmany	assess the impact of an integrated health care project on perceived patient participation in medical decision-making	quasi-experimental controlled cohort-study	primary integrative care	SDM-Q-9 (assessment not directly after consultation)	primary	training for physicians	chronically ill patients
Körner et al. (2012), Germany	evaluate an interprofessional SDM training	cRCT	inpatient specialty care	SDM-Q-9 (adaptation for all HCPs, assessment point n/r)	primary	interprofessional training programme	n/r
Körner et al. (2014), Germany	evaluate an interprofessional SDM training programme	cRCT	inpatient specialty care	SDM-Q-9 &-Doc (adaptation for all HCP’s perspectives, directly after consultation)	primary	interprofessional training programme	n/r
Tinsel et al. (2013), Germany	implement and evaluate a SDM training programme for GPs on perceived participation	cRCT	primary care	SDM-Q-9 (directly after consultation)	primary	training for GPs	hypertension

GP = general practitioner, DA = decision aid, HCP = health care provider, n/r = not reported;

* only objectives which could be answered with SDM-Q-9 &/-Doc

**Table 3:** Characteristics of the included original studies (continued).

**First author (year), country**	**measurement points**	**patients (N, age (mean),women)+**	**HCP (profession, N, age, women)+**	**SDM-Q-9 &/-Doc scores (mean, SD)**	**Results (effects–mean diff., significance)**
Brito et al. (2015), USA	T0 = CG, TAU, pre-intervention, T1 = implementation	N = 51 age 42.8, women: IG 81%, CG 78%	endocrinologists; N = 9; Age and gender n/r	IG: patients:20(19,21)^1^, physicians:20(18,23)^1^; CG: patients:19(17,21)^1^ physicians:19(17,21)^1^	no significant effects; mean-diff. IG-CG^2^: patients: 0.99 (CI 95%, -0.98, 3.0) p = 0.47; physicians: 1.4 (CI 95%, -1.5, 4) p = 0.18
Hölzel et al, (2012), Germany	T0 = baseline, T1 = implementation, T2 = implementation	N = 2188, age: IG: 62,9, CG1: 62,8, CG2: 63,3; women: IG: 59,1%, CG1: 59,3%, CG2: 58,7%	GPs; N, age and gender n/r	IG: T0 = 72,4 (24,2),T1 = 68,9 (24,9),T2 = 65,8 (28,1); CG1: T0 = 71,1 (25,3), T1 = 68,7 (24,5),T2 = 69,2 (26,7); CG2: T0 = 69,0 (25,1), T1 = 67,7 (25,0), T2 = 66,1 (28,3)	no significant effect for intervention, experienced involvement decreased over time: p < 0,01, independently of group (IG) p = 0,31 (no significant interaction-effect: p = 0,17), statistical power: due to sample size, ƞ^2^ = 0,01
Körner et al. (2012), Germany	T0 = pre-intervention, T1 = post-intervention	not applicable	physicians; nurses; psycho- social therapists, physical therapists; other N = 179;age: 36 to 55 y; women: 64,8%; IG: 56,5%, CG: 70%	IG: T0 = 63.7 (21.6), T1 = 75.2 (12.4); CG: T0 = 67.9 (21.1), T1 = 67.7 (22.5)	no significant effect overall mean-diff.: pre-post: F period x group (1) = 2.806, p = .095, ƞ^2^ = .008), occupational groups: Focc. group (4) = 8.372, p < .001, ^2^ = .089)
Körner et al. (2014), Germany	T0 = pre-intervention, T1 = post-intervention, T2 = 6 months follow-up	N = 1419; age: IG: 57.1, CG: 53.6 women: IG: 40,6%, CG: 33,1%	physicians, nursing staff, physical therapists, sport teachers, masseur, psychologists, other psychosocial therapists, dietitians, social workers; N = 662, age: 36–55 y.; women: IG: 52.4%, CG: 61,9%	patient-survey: IG: T0 = 55.6 (26.2), T1 = 57 (26.4), T2 = 57.5 (26.4), CG: T0 = 59.1 (26.3), T1 = 59 (25.2),T2 = 58.3 (27.7) staff-survey: IG: T0 = 62.5 (22), T1 = 72.9 (17.3); CG: T0 = 67.2 (21.6), T1 = 67.3 (22.5)	small significant intervention-effect for staff, CG remained unchanged, mean-diff.: staff: F group x period: p = 0.028, ƞ^2^ = .014
Tinsel et al. (2013), Germany	T0 = pre-intervention, T1 = 6 months follow-up, T2 = 12 months follow-up, T3 = 18 months follow-up	N = 1120; age: IG: 63.8, CG: 65.0; women: IG: 53,3%, CG: 55,3%	GPs; N = 37; Age and gender n/r	IG: T0 = 73.00 (17.66), T1 = 73.03 (19,54),T2 = 70.51 (20,98), T3 = 71.71 (20.59) CG: T0 = 70.67 (20.24), T1 = 66.55 (21.34), T2 = 67,20 (20.00), T3 = 66.60 (20.71)	no significant effect for intervention on perceived participation; mixed model analysis: change from T0 was 3.11 points higher in IG com-pared to CG, 97,5% CI, (-2,37; 8,61), p = 0.203

GP = general practitioner HCP = health care professional, IG = intervention group, CG = control group, TAU = treatment as usual, CI = confidence interval, SD = standard deviation, p = p-value

^1^ = raw score from 0 (lowest) - 45 (highest); a transformation of a raw score of 20 leads to a sum score of 44.4 and to 42.2 for a raw score of 19

+ sample characteristics of first measurement point, n/r = not reported

### Description of included study protocols

The description of the included study protocols can be viewed in Table 4 and Table 5. Four of six protocols described cRCTs [49–52]. Three studies are planned to be conducted in Germany [50–52]. The studies will be conducted in various health care settings. Two of six studies will use both SDM-Q-9 and SDM-Q-Doc [49, 53]. There will be one adaptation of the instrument for a patient’s companion [54] and one for an observer’s perspective [49]. One study protocol reported an assessment of SDM-Q-9 directly after the clinician-patient-consultation [54]. Two studies will assess the SDM-Q-9 as primary outcomes [49, 53]. There will be different forms of interventions, decision aids, and trainings, and most will aim both at physicians and patients [49, 50, 52–54]. While all six studies will have clustering on the clinic- or practice-level, three took clustering into account in their reported sample size calculation [49, 50, 52] and two in their planned statistical analyses [50, 51]. For the complete data extraction sheet of study protocols please see S7 Table.

**Table 4:** Characteristics of the included study protocols.

**First author (year), country**	study objectives*	**study design**	**health care setting**	**SDM-Q-9 &/-Doc (assessment point)**	**primary or secondary outcome**	**intervention**	decisional context
den Ouden et al., (2015), Netherlands	evaluate if a DA increases SDM	cRCT	primary care	SDM-Q-9 &-Doc (& adaptation for observer; assessment point n/r)	primary and secondary	DA & training	Type 2 Diabetes
Drewelow et al., (2012), Germany	evaluate if intervention is able to increase SDM	cRCT	primary care	SDM-Q-9 (assessment via phone calls)	secondary	PC based- DA, 2 group-trainings after peer-visit	Type 2 Diabetes Mellitus
Geiger et al., (2011), Germany	evaluate if intervention improves SDM	cRCT	outpatient specialty care	SDM-Q-9 (assessment point n/r)	secondary	video feedback based training & manual	n/r
Goss et al., (2015), Italy	evaluate a pre-consultation intervention to increase involvement in consultation	RCT	outpatient specialty care	SDM-Q-9 (adaptation for companion; directly after consultation)	secondary	question prompt sheet	breast cancer
Löffler et al., (2014), Germany	evaluate the effectiveness of an intervention to reduce the number of long-term drugs	cRCT	inpatient primary & secondary** care	SDM-Q-9 (data collection at admission & phone call)	secondary	narrative-based medication review	chronic diseases, multimorbidity & polypharmacy
Savelberg et al, (2015), Netherlands	evaluate impact of DA on SDM	pre-/post-implementation study	inpatient specialty care	SDM-Q-9 &-Doc (assessment point n/r)	primary	DA website & training for HCP	surgical treatment of breast cancer

HCP = health care professional, DA = decision aid

* only objectives which could be answered with SDM-Q-9 &/-Doc

** aftercare, n/r = not reported

**Table 5:** Characteristics of the included study protocols (continued).

**First author (year), country**	**measurement points**	**recruitment, HCP**	**sample-size calculation (ICC)**
den Ouden et al., (2015), Netherlands	T0 = pre-intervention T1 = 12 months follow-up T2 = 24 months follow-up	79 general practices (GPs)	N = 73 per group, p = 80%, α 0.05, CI = 95% —> ICC [1 (m-1)r] r = 0.025
Drewelow et al., (2012), Germany	T0 = pre-intervention, T1 = 6 months follow-up, T2 = 12 months follow-up, T3 = 18 months follow-up, T4 = 24 months follow-up	20 GPs per study centre (13 patients/ practice)	54 GPs with 13 patients, N = 780 patients (derived factor 1.9, ICC 0.1, average cluster size of 10; p = 80%)
Geiger et al., (2011), Germany	T0 = pre-intervention, T1 = IG: intermediate, CG: waiting assessment, T2 = IG: post-intervention, CG: intermediate assessment, T3 = IG: 6 months follow-up CG: post-intervention	7 university outpatient clinics, oncologists, gynaecologists, psychiatrists, neurologists, dentists, radiologists	N = 76 patients, (α 0.05, p = .85), N = 36 physicians (α 0.05, p = .85) —> no ICC reported
Goss et al., (2015), Italy	T1 = directly after consultation (SDM-Q-9 not at baseline)	3 oncology depart-ments, oncologists	N = 260 patients, 130 per group (p = 80%, α 0.05) —> no ICC reported
Löffler et al., (2014), Germany	T0 = pre-intervention, (admission to hospital), T1 = discharge from hospital, T2 = 6 months follow-up, T3 = 12 months follow-up	4 clinics (30 patients/ week& clinic), pharmacists (GPs & hospital physicians)	N = 1544 patients (p = 80%, α 0.05), IG: 772 & CG: 772 in 42 wards—> with ICC 0.1
Savelberg et al, (2015), Netherlands	T0 = pre-implementation, T1 = implementation, T2 = post-implementation	4 hospitals, breast surgeons, radiation oncologists, nurses	T0 & T1: N = 10, T2: N = 4 per hospital from implementation sample (N = 16)

GP = general practitioner, IG = intervention group, CG = control group, ICC = intracluster correlation coefficient

### Methodological quality of included original studies

In summary, four original studies were rated “poor” [44–47] and one was rated as “fair” [48] (see S1 to S3 Tables).

The drop-out rate of the intervention gsroup participants exceeded 20% in all controlled intervention studies, which is viewed as a ‘fatal flaw’, resulting in a “poor” rating [44–46] (S1 Table). The randomisation process was described in one study [45]. Neither of the studies conducted independent recruitment of participants or blinding of HCPs. The differential drop-out rate between intervention and control group was over 15% in two studies [44, 45], which is also considered a ‘fatal flaw’. Data on adherence to the intervention protocol or the utilization of other interventions were not reported [44–46]. Furthermore, none of the three cluster randomised trials reported a sufficiently large sample size necessary for detecting effects with ≥80% power [44–46]. One study controlled for baseline imbalances, took clustering effects into account in sample size calculation and statistical analysis of endpoints, and also explicitly reported an intention-to-treat analysis [46].

The quality of the implementation study with a historical control group was rated “poor” (S2 Table) as neither blinding of participants nor multiple times of measurement were reported. In addition, the intervention was not delivered consistently across the study population. All other criteria could be answered with “Yes”.

The quasi-experimental controlled cohort study [48] received an overall “fair”-rating (S3 Table). The participation rate of eligible persons was <50% and the loss to follow-up after baseline >20%. Criteria 6, 8, 9 and 10 were rated as not applicable. Blinding of the outcome assessors was not reported. All other criteria were fulfilled.

### Quality of included study protocols

In summary five study protocols were rated as “fair” [49–53] and one as “good” [54] (see S4 Table and S5 Table).

The assessment tool for *controlled intervention studies* was utilized for one RCT-protocol [54] which received a “good” rating and four cRCT-protocols [49–52] which were rated “fair” (S4 Table). One cRCT-protocol did not use the term “cluster” in the description of the study design, did not take cluster-effects into account in the sample size calculation, and did not pre-specify outcomes [51]. Two out of five protocols did not report randomisation processes [49, 50], and three did not report on allocation concealment [49, 50, 54]. Blinding of participants was planned by one protocol [51], while two others did not report on this [50, 54]. Blinding of HCPs was planned in two studies [51, 54]. One of four cRCT-protocols reported independent recruitment of participants, [52] and one planned blinded assessment of outcomes [51]. Two protocols reported plans to ascertain baseline similarities of samples [51, 54] whereas one cRCT-protocol planned adjustment for baseline imbalances [52]. No protocol addressed utilization of other interventions. All study protocols included a sample size calculation and all four cRCTs regarded cluster effects in planned statistical analyses. All but one study protocol [54] planned analyses according to the intention-to-treat principle.

The protocol of a pre-post-implementation study with a historical control group [53] received a “fair” rating, (S5 Table) as no planned inference statistics were reported and the measurement of outcome variables was not planned for multiple times before and after implementation of the intervention. Furthermore, there was no information on blinding of people assessing outcomes. All other criteria were fulfilled.

## Discussion

This systematic review aimed to 1) examine the use of the SDM-Q-9 and -Doc in intervention studies on SDM in clinical settings, 2) describe how the SDM-Q-9 and–Doc performed regarding sensitivity to change, and 3) assess the methodological quality of studies and study protocols using the measure. Five studies and six study protocols were included in this review.

Most reported trials were conducted in Europe. Four studies used both the SDM-Q-9 and SDM-Q-Doc [45, 47, 49, 53], whereas all others used the SDM-Q-9 only. In four trials the measure was adapted for other participants [44, 45, 49, 54], and seven of the included trials used it to assess primary outcomes. [44, 45, 47–49, 53]. Our results reveal a range of the measure’s application areas, although many studies assessed SDM in primary care settings [46, 48–50]. Moreover, the SDM-Q-9 and -Doc was applied to evaluate diverse interventions facilitating SDM, but was mainly used to assess training programs for HCPs and/or decision aids.

The reported mean sum scores ranged from 42 to 75 on a scale from 0 to 100. There were no significant changes detected in the mean-differences between intervention and control groups in four of five studies, and the detected difference in the fifth study [45] was small in size. This could hint at deficiencies of the sensitivity to change of the SDM-Q-9 and -Doc. However, several other explanations for this finding are also possible. First, the duration of the evaluated interventions was only reported by two studies [46, 48], both of which were relatively brief. The intervention dose might have been too little to accomplish behavior change. Research shows various barriers that need to be addressed for successful changes in behavior [55–57]. Positive attitude towards SDM do not automatically result in implementation into practice [58]. Furthermore, interventions targeting both patients and HCPs have been found to be more effective than single-target interventions. Thus, it is possible that some interventions did not succeed in implementing SDM. Second, two studies did not report direct assessment of the SDM-Q-9 and -Doc after the relevant consultation [44, 48], which leaves room for bias of effects by uncontrolled influences. Third, few original studies described the HCP sample characteristics, and they did not control for those variables athough there is evidence of their influence on SDM [59]. Thus, the results of this review do not allow us to draw firm conclusions on the measure’s sensitivity to change. A psychometric study focusing on the measure’s sensitivity to change is strongly recommended. Such a study could also investigate whether response formats other than the present 6-point Likert scale, can increase sensitivity to change.

Study quality, as measured by the Quality Assessment Tools from the U.S. National Institute of Health, was assessed for seven cRCTs, one RCT, two pre and post-implementation studies and one quasi-experimental controlled cohort study. Of the original studies, only the quasi-experiment was rated “fair” with some risk of bias [48]. All others received a “poor” rating, as they had ‘fatal flaws’ with high risk of bias to their internal validity [44–47]. Admittedly, the “fair” rating has to be handled with caution, as the quality assessment instrument did not completely fit the study design. Quality of the rated study protocols was slightly better, with five “fair” ratings [49–53] and one “good” rating for the RCT [54] with very low risk of bias. This might be due to the fact that not all items could be applied to those trials. As protocols do not contain results, they leave less room for possible flaws, especially as many original studies were rated poor due to a high drop-out rate, which cannot be rated for study protocols. Even so, there was a great difference in detail and completeness of methodological description between study protocols and original studies. This could also be explained by gradually higher adherence to research and reporting guidelines over time, leading to slightly better rating for the more recent study protocols. Still, even the more detailed methodology descriptions of protocols did not always satisfy the criteria regarding randomisation. The definition of ‘fatal flaw’ as high drop-out rate (>20% drop-out at endpoint in the intervention group) might be unlikely to be fulfilled in health care research studies under routine conditions. Especially in primary care, many factors aside from intervention effects could influence follow-up rates, as there are practical reasons for changing one’s general practitioner (e.g. move into another area). The difficulty of blinding HCPs to treatments when evaluating trainings in SDM for HCPs should also be taken into account. The criteria from the risk of bias tool for *observational cohort and cross-sectional studies* demanding 50% participation of the eligible population and ≤20% loss to follow-up after baseline seems difficult to achieve considering clinical care population sizes, return rates of postal recruitment and repeated measurements. For example, Tinsel et al. (2013) report that loss to follow-up was generally higher in primary care studies with long-term follow-up [46]. In many of the included studies recruitment was done by the general practitioner (GP). However, recruitment by GPs is found to be less successful and trials’ general success might even decrease if the GPs’ alertness during consultations is essential [60], which is undoubtedly the case for SDM. Consequently, ratings of original studies might have been better with less strict criteria. Despite the range of factors that can explain the quality ratings, the overall quality of included intervention studies must be summarised as tenuous and the quality of intervention study protocols as moderate.

The SDM-Q-9 and -Doc are relatively young instruments, and translating them, conducting a study, and publishing data take time. Some excluded articles in our screenings still used the first version from 2006 [61]. More research with the measure is underway, so feedback from different researchers and results from the included protocols are yet to come. There were more than twenty articles found in the screenings that utilised the measure for other purposes, such as validating new SDM measures or simply to assess the status of SDM in a clinical setting. An update of the present systematic review in a couple of years would certainly be helpful to draw better conclusions from a larger number of studies on the measure’s sensitivity to change.

There are several strengths and limitations of the present systematic review. One strength is a comprehensive database search combined with a comprehensive secondary search. Another strength is that the title and abstract screenings as well as the full text screenings were done by two independent reviewers for all articles. The same applies to the conducted quality assessment. A main limitation is that the data extraction was performed by only one reviewer, which lends room to possible bias. It must be noted that only results of five completed studies could be assessed, which might decrease the generalisability of the review’s conclusions. Furthermore, this review focused on adult patients, mainly because the 9-item Shared Decision Making Questionnaire is designed for use in adult populations. However, the use of SDM in pediatric populations is a growing area of clinical and research interest. Thus, the adaptation of the measure for use in this setting could also be an area of future research.

In conclusion, the identified records showed a range of the measure’s application in different health care settings and its use to evaluate diverse interventions. We found the included studies to be of limited methodological quality. Our results also suggest that future articles on original studies should describe the methodology and interventions in more detail. Research ought to assess HCP characteristics more thoroughly, conduct independent recruitment, and control for actual implementation of SDM. Future trials ought to either contemplate randomisation at patient-level, or correct for clustering effects in cRCT sample size calculations and statistical analyses. The SDM-Q-9 and -Doc’s sensitivity to change remains unclear. It is uncertain whether the measure does not assess changes or if there were no changes in perceived SDM. Therefore, it might be advisable to combine the SDM-Q-9 and -Doc with an observer based measure of SDM, as Scholl and colleagues have found that the patient-reported measure does not correlate significantly with an observer-based instrument [62]. Likewise, a combination with instruments assessing actual change in patient and HCP behavior regarding SDM in future studies seems reasonable. The heterogeneity of trials examining interventions facilitating SDM is vast and makes comparisons and examination of perceived SDM difficult.

This review may help researchers decide whether the measure fits their purposes. Furthermore, it shows risks of bias in previous trials which used the measure and may help prospective researchers to decrease these risks. Also, more research on the measure’s sensitivity to change is strongly suggested before using it in further intervention studies.

## Supporting information

S1 Appendix Electronic data base search strategy for EMBASE, PsycINFO, Medline. NR = not reported, NA = not applicable; sources for added criteria: 1.1 [51], 4.1 [52, 53], 6.1 [51, 54–57], 12.1 a) & b) [51, 57–59], + To understand the trial procedure of Körner et al. 2014, the article of Körner et al. 2012 needed to be consulted as the description of methodology and terminology was otherwise unclear for two reviewers.
(DOCX)

S1 Table Quality assessment of controlled intervention studies (original studies). (DOCX)

S2 Table Quality assessment for before-after studies (original studies). (DOCX)

S3 Table Quality assessment for observational cohort and cross-sectional studies (original studies). NR = not reported, NA = not applicable.
(DOCX)

S4 Table Quality assessment of controlled intervention studies (study protocols). CD = cannot determine, NR = not reported, NA = not applicable; sources for added criteria: 1.1 [51], 4.1 [52, 53], 6.1 [51, 54–57], 12.1 a) & b) [51, 57–59].
(DOCX)

S5 Table Quality assessment for before-after-studies (study protocols). NR = not reported
(DOCX)

S6 Table Data extraction sheet for original studies. (DOCX)

S7 Table Data extraction sheet for study protocols. (DOCX)

S8 Table Prisma checklist. (DOCX)
