# Supplementary material for: Use of the 9-item Shared Decision Making Questionnaire (SDM-Q-9 and SDM-Q-Doc) in intervention studies—A systematic review
Source: PLoS One. 2017 Mar 30;12(3):e0173904. doi: 10.1371/journal.pone.0173904 (PMC5373562; doi:10.1371/journal.pone.0173904)
Supplement: S1 Appendix — NR = not reported, NA = not applicable; sources for added criteria: 1.1 [51], 4.1 [52, 53], 6.1 [51, 54–57], 12.1 a) & b) [51, 57–59], + To understand the trial procedure of Körner et al. 2014, the article of Körner et al. 2012 needed to be consulted as the description of methodology and terminology was otherwise unclear for two reviewers. (DOCX) [file pone.0173904.s001.docx]

**S1 Appendix. Electronic Data Base Search Strategy for EMBASE, PsycINFO, Medline.**

**EMBASE via Ovid (English):**

SDM-Q-9 (OR SDM-Q-Doc / SDM-Q / SDM Questionnaire / Nine-item shared decision making questionnaire / 9-item shared decision making questionnaire / shared decision making questionnaire)

Limitations: 2010 to current

**EMBASE via Ovid (German):**

Fragebogen zur partizipativen Entscheidungsfindung (OR PEF-FB / PEF-FB-9 / PEF-FB-Doc / Fragebogen partizipative Entscheidungsfindung)

Limitations: 2010 to current

**PsycINFO via Ovid:**

SDM-Q-9 (OR SDM-Q-Doc / SDM-Q / SDM Questionnaire / Nine-item shared decision making questionnaire / 9-item shared decision making questionnaire / shared decision making questionnaire)

Limitations: 2010 to current

**Medline via Ovid:**

SDM-Q-9 (OR SDM-Q-Doc / SDM-Q / SDM Questionnaire / Nine-item shared decision making questionnaire / 9-item shared decision making questionnaire / shared decision making questionnaire)

Limitations: 2010 to current

Electronic database searches were conducted on the 13^th^ October 2015.
