# Supplementary material for: Use of the 9-item Shared Decision Making Questionnaire (SDM-Q-9 and SDM-Q-Doc) in intervention studies—A systematic review
Source: PLoS One. 2017 Mar 30;12(3):e0173904. doi: 10.1371/journal.pone.0173904 (PMC5373562; doi:10.1371/journal.pone.0173904)
Supplement: S1 Table — (DOCX) [file pone.0173904.s002.docx]

**S1 Table. Quality Assessment of Controlled Intervention Studies (Original Studies).**

| Quality Assessment of controlled Intervention Studies | Körner et. al 2012 | Körner et. al 2014 | Tinsel et. al 2013 |
| --- | --- | --- | --- |
| 1. Was the study described as randomised, a randomised clinical trial, or an RCT? | NA | NA | NA |
| 1.1 Or did they describe it as cluster randomised? | Yes | Yes | Yes |
| 2. Was the method of the randomisation adequate (i.e., use of randomly generated assignment)? | Yes | NR | NR |
| 3. Was the treatment allocation concealed (so that assignments could not be predicted)? | Yes | NR | NR |
| 4.a) Were study participants blinded to the treatment-group assignments? | Yes | NR | Yes |
| 4. b) Were providers blinded to the treatment group assignments? | No | NR | No |
| 4.1 In case of cluster-randomisation: Was the recruitment of participants conducted by an individual independent of the trial? | No | NR | No |
| 5. Were the people assessing the SDM-Q-9 &/ -Doc blinded to the participant’s group assignment? | No | NR | Yes |
| 6. Were the groups similar at baseline on important characteristics that could affect outcomes (i.e., demographics, risk-factors, co-morbid conditions)? | No | No | Yes |
| 6.1 In case of cluster randomisation: Did they use stratification or matched-pairs before randomisation to reduce baseline-imbalances? | No | No | No |
| 7. Was the overall drop-out rate from the study at endpoint 20% or lower of the number allocated to treatment? | No | No | No |
| 8. Was the differential drop-out rate (between treatment groups) at endpoint 15 percentage points or lower? | No | No | Yes |
| 9. Was there high adherence to the intervention protocols for each treatment group? | NR | NR | NR |
| 10. Were other interventions avoided or similar in the groups (e.g., similar background treatments)? | NR | NR | NR |
| 12. Did the authors report the calculation of a sufficiently large sample size to be able to detect a difference in the main outcome between groups with at least 80% power? | No | No | No |
| 12.1 a) In case of cluster-randomisation: Did they take clustering effects into account in their statistical analysis? | No | No | Yes |
| 21.1 b) In case of cluster-randomisation: Did they consider intra-class-correlation regarding sample size calculation? | No | No | Yes |
| 13. Were outcomes or analysed subgroups which were reported prespecified? (i.e., identified before analyses was conducted)? | Yes | Yes | Yes |
| 14. Were all randomised participants analysed in the group to which they were originally assigned, i.e., did they use an intention-to-treat analysis? | NR | NR | Yes |
| Quality rating: (good, fair or poor) | poor | poor | poor |
