# Supplementary material for: Use of the 9-item Shared Decision Making Questionnaire (SDM-Q-9 and SDM-Q-Doc) in intervention studies—A systematic review
Source: PLoS One. 2017 Mar 30;12(3):e0173904. doi: 10.1371/journal.pone.0173904 (PMC5373562; doi:10.1371/journal.pone.0173904)
Supplement: S2 Table — (DOCX) [file pone.0173904.s003.docx]

**S2 Table. Quality Assessment for Before-After Studies (Original Studies).**

| Quality Assessment for Before-After (Pre-Post) Studies With No Control Group | Brito et. al, 2012 |
| --- | --- |
| 1. Was the study question or objective clearly stated? | Yes |
| 2. Were eligibility/selection criteria for the study population prespecified and clearly described? | Yes |
| 3. Were the participants in the study representative of those who were eligible for the test/service/intervention in the general or clinical population of interest? | Yes |
| 4. Were all eligible participants that meet the prespecified entry criteria enrolled? | Yes |
| 5. Was the sample size sufficiently large to provide confidence in the findings? | Yes |
| 6. Was the test/service/intervention clearly described and delivered consistently across the study population? | No |
| 7. Were the outcome measures prespecified, clearly defined, valid, reliable and assessed consistently across all study participants? | Yes |
| 8. Were the people assessing the outcomes blinded to the participants' exposure/interventions? | No |
| 9. Was the loss to follow-up after baseline 20% or less? Were those lost to follow-up accounted for in the analysis? | Yes |
| 10. Did they use statistical methods that examined changes in outcome measures from before to after the intervention? Were statistical tests done that provided p values for the pre-to-post changes? | Yes |
| 11. Were outcome measures of interest taken multiple times before the intervention and multiples times after the intervention (i.e., did they use an interrupted time-series design)? | No |
| 12. If the intervention was conducted at a group level (e.g., a whole hospital, a community, etc.) did the statistical analysis take into account the use of individual-level data to determine effects at the group level? | Yes |
| Quality rating (good, fair, poor): | poor |
