# Supplementary material for: Use of the 9-item Shared Decision Making Questionnaire (SDM-Q-9 and SDM-Q-Doc) in intervention studies—A systematic review
Source: PLoS One. 2017 Mar 30;12(3):e0173904. doi: 10.1371/journal.pone.0173904 (PMC5373562; doi:10.1371/journal.pone.0173904)
Supplement: S4 Table — CD = cannot determine, NR = not reported, NA = not applicable; sources for added criteria: 1.1 [51], 4.1 [52, 53], 6.1 [51, 54–57], 12.1 a) & b) [51, 57–59]. (DOCX) [file pone.0173904.s005.docx]

**S4 Table. Quality Assessment of Controlled Intervention Studies (Study Protocols).**

| Quality Assessment of controlled Intervention Studies | den Ouden et. al 2015 | Drewelow et. al 2012 | Geiger et. al 2011 | Goss et. al 2015 | Löffler et. al 2014 |
| --- | --- | --- | --- | --- | --- |
| 1. Is the study described as randomised, a randomised clinical trial, or an RCT? | NA | NA | Yes | Yes | NA |
| 1.1 Or do they describe it as cluster randomised? | Yes | Yes | No | NA | Yes |
| 2. Is the method of the randomisation adequate (i.e., use of randomly generated assignment)? | NR | NR | Yes | Yes | Yes |
| 3. Is the treatment allocation concealed (so that assignments could not be predicted)? | NR | NR | CD | Yes | Yes |
| 4.a) Will study participants be blinded to the treatment-group assignments? | No | NR | Yes | NR | No |
| 4. b) Will providers be blinded to the treatment group assignments? | No | NR | Yes | Yes | No |
| 4.1 In case of cluster-randomisation: Will the recruitment of participants be conducted by an individual independent of the trial? | No | No | NR | NA | Yes |
| 5. Will the people assessing the SDM-Q-9 &/ -Doc be blinded to the participant’s group assignment? | No | NR | Yes | NR | No |
| 6. Will the groups be similar at baseline on important characteristics that could affect outcomes (i.e., demographics, risk-factors, co-morbid conditions)? | NR | NR | Yes | Yes | NA |
| 6.1 In case of cluster randomisation: Will they use stratification or matched-pairs before randomisation to reduce baseline-imbalances? | No | No | No | NA | Yes |
| 10. Will other interventions be avoided or similar in the groups (e.g., similar background treatments)? | NR | NR | NR | NR | CD |
| 12. Do the authors report the calculation of a sufficiently large sample size to be able to detect a difference in the main outcome between groups with at least 80% power? | Yes | Yes | Yes | Yes | Yes |
| 12.1 a) In case of cluster-randomisation: Will they take clustering effects into account in their statistical analysis? | Yes | Yes | Yes | NA | Yes |
| 21.1 b) In case of cluster-randomisation: Will they consider intra-class-correlation regarding sample size calculation? | Yes | Yes | No | NA | Yes |
| 13. Are outcomes or analysed subgroups which will be reported prespecified? (i.e., identified before analyses was conducted)? | Yes | Yes | No | Yes | Yes |
| 14. Will all randomised participants be analysed in the group to which they are originally assigned, i.e., do they use an intention-to-treat analysis? | Yes | Yes | NR | Yes | Yes |
| Quality rating: (good, fair or poor) | fair | fair | fair | good | fair |
