# Supplementary material for: Use of the 9-item Shared Decision Making Questionnaire (SDM-Q-9 and SDM-Q-Doc) in intervention studies—A systematic review
Source: PLoS One. 2017 Mar 30;12(3):e0173904. doi: 10.1371/journal.pone.0173904 (PMC5373562; doi:10.1371/journal.pone.0173904)
Supplement: S5 Table — NR = not reported (DOCX) [file pone.0173904.s006.docx]

**S5 Table. Quality Assessment for Before-After-Studies (Study Protocols).**

| Quality Assessment for Before-After (Pre-Post) Studies With No Control Group | Savelberg et. al 2015 |
| --- | --- |
| 1. Is the study question or objective clearly stated? | Yes |
| 2. Are eligibility/selection criteria for the study population be prespecified and clearly described? | Yes |
| 3. Will the participants in the study be representative of those who were eligible for the test/service/intervention in the general or clinical population of interest? | Yes |
| 4. Will all eligible participants that meet the prespecified entry criteria be enrolled? | Yes |
| 5. Will the sample size be sufficiently large to provide confidence in the findings? | Yes |
| 6. Will the test/service/intervention be clearly described and delivered consistently across the study population? | Yes |
| 7. Will the outcome measures be prespecified, clearly defined, valid, reliable and assessed consistently across all study participants? | Yes |
| 8. Will the people assessing the outcomes be blinded to the participants' exposure/interventions? | NR |
| 10. Will they use statistical methods that examine changes in outcome measures from before to after the intervention? Will statistical tests be done that provided p values for the pre-to-post changes? | No |
| 11. Will outcome measures of interest be taken multiple times before the intervention and multiples times after the intervention (i.e., will they use an interrupted time-series design)? | No |
| 12. If the intervention was conducted at a group level (e.g., a whole hospital, a community, etc.) will the statistical analysis take into account the use of individual-level data to determine effects at the group level? | No |
| Quality rating (good, fair, poor): | Fair |
