# Supplementary material for: Use of the 9-item Shared Decision Making Questionnaire (SDM-Q-9 and SDM-Q-Doc) in intervention studies—A systematic review
Source: PLoS One. 2017 Mar 30;12(3):e0173904. doi: 10.1371/journal.pone.0173904 (PMC5373562; doi:10.1371/journal.pone.0173904)
Supplement: S6 Table — (DOCX) [file pone.0173904.s007.docx]

**S6 Table. Data Extraction Sheet for Original Studies.**

| Study, year of publication: | Brito et. al 2015 | | Hölzel et. al 2012 | |
| --- | --- | --- | --- | --- |
| authors | Brito J.P., Castaneda-Guarderas A., Gionfriddo M.R., Ospina N.S., Maraka S., Dean D.S., Castro R.M., Fatourechi V., Gharib H., Stan M.N., Branda M., Bahn R.S., Montori V. M. | | | Hölzel L.P., Vollmer M., Kriston L., Siegel A., Härter M. |
| title | Development and pilot testing of an encounter tool for shared decision making about the treatment of Graves' Disease | | | Patientenbeteiligung bei medizinischen Entscheidungen in der  Integrierten Versorgung Gesundes Kinzigtal: Ergebnisse einer kontrollierten Kohortenstudie |
| study aim & research questions | develop & test an encounter decision tool (Grave's Disease Choice) for patients and clinicians to engage in SDM about the treatment of Grave's Disease | | | assess the impact of an integrated health care project called  "Gesundes Kinzigtal" (GK) on perceived patient participation in medical decision making (as well as preferred involvement & information, decision confidence, satisfaction with out-patient care and physical & psychological quality of life) |
| country | U.S.A. - Rochester, Minnesota | | | Germany |
| primary/specialty care in-/out-patient care | specialty care out-patient care | | | primary care out-patient - integrative care |
| medical condition/specific decisional context (treatments) | Grave's Disease (Morbus Basedow) 1) anti-thyroid drugs, 2) radioiodine ablation, 3) surgery | | | patients having to decide which therapy; aiming at chronically diseased, or patients with risk of developing a chronic disease --> no specific treatments |
| instrument version used (1=SDM-Q-9, 2= SDM-Q-Doc, 3= both) | 3 | | | 1 |
| adaptation of instrument (form) | not reported | | | not reported |
| language/translation | English | | | German |
| former psychometrical testing | not reported | | | not reported |
| study type (e.g. control group, randomisation[level], stratification, blinding?) | historically controlled pre-/post implementation study (no randomisation, no stratification, no blinding) | | | quasi-experimental controlled cohort-study (with IG and 2 CGs, natural cohorts, stratification of sample, no blinding) |
| points of measurement (SDM-Q-9 &/-Doc directly after treatment?) | T0 = TAU (CG), Apr. to Dec. 2013, pre-intervention, T1 = implementation, Jan. 2014 to Mar. 2015, further: video recordings of encounter, rated by observers, --> questionnaires given directly after encounter, answering-mechanism not reported | | | T0 = 2007, baseline (implementation),T1 = 2008 (implementation), T2 = 2009 (implementation) --> questionnaires distributed via post, (not directly after consultation) |
| use of SDM-Q-9 &/-Doc as primary/secondary outcome | primary | | | primary |
| other outcomes assessed (1= primary, 2= secondary outcome) & which points of measurement? | no further differentiation on primary/secondary outcomes reported; decisional conflict ("uncertainty" subscale of Decisional Conflict Scale); patient's knowledge about treatment options for DG (self-made questionnaire); extent to which clinicians engaged patients in the decision making process, evaluating video-recordings (Observer OPTION Scale), fidelity to the DA (self-made checklist to assess the clinicians use of DA as intended, for video/audio recording) | | | 2) preference for involvement & information (Autonomie-Präerenz-Index,API), 2) confidence in decision made (Decisional Conflict Scale, DCS), 2) satisfaction with medical care (questionnaire of satisfaction in out-patient care, Zufriedenheit mit der ambulanten Versorgung, ZAPA), 2) bodily & physical quality of life (SF-12; short form of Health Survey FSF-36) |
| if both versions used: did they measure the same situation? | yes | | | not applicable |
| handling of missing data SDM-Q-9 &/-Doc (Intention-to-Treat principle? ITT) | SDM-Q-9 &-Doc: imputation by at least 2 missing items; (mean score imputed) | | | ITT, Expecto-Maximization-Algorithm: 22,3% of all data imputed  (mostly missing in T1 & due to low acceptance of SF-12 & DCS); (no highly explained variance of non-responding by group variables found) |
| treatment as usual (TAU) | consultation as usual (before implementation of DA) | | | 1) not being part of the integrative care system GK, 2) living in another comparable rural area without an integrative care system |
| description of intervention (patient-&/practitioner-level, goal, form & duration) | no description of DA given"GD Choice", version in appendices goal of DA: support a conversation between patient and doctor in which patients verbalize intent to "trying on" the different options (reference of 2 other papers about development of DA given) | | | "Gesundes Kinzigtal"-project:  integrative care system for primary care patients; goal: higher patient involvement and satisfaction with the medical care system; SDM specific: three 3 hour trainings of clinicians which were part of the project (2008 and 2009) |
| Recruitment (for cluster-randomisation: independent recruiter?) | Thyriod Clinic within the Division of Endocrinology at the Mayo Clinic  in Rochester, Minnesota | IG: patients who were part of the GK-project - full sample survey of patients from AOK/LKK Baden-Würtemberg at 31st July 2007  CG1: patients who were not part of it ; CG2: patients from another comparable area without an integrative care system; | | |
| Size & power-calculation (ICC) | N = 60, at least 30 per group (power 80%, to detect minimum 10 points, alpha 0.05) | N = not reported, power calculation given (ƞ² = 0,01 with 99% probability) | | |
| inclusion criteria | adults with GD who needed treatment for this condition & gave consent to participate in the study; IG additionally: patients who had not been seen in the previous 9 months | IG: AOK/LLK health insurance, taking part in the GK project; CG1: AOK/LLK health insurance, not taking part in the GK project; CG2: AOK/LLK health insurance, not part of an integrative system, living in Sigmaringen/Biberach (comparable area) | | |
| exclusion criteria | pregnant women patients were the risks & benefits of one/more treatment options were thought to be significantly different than expected from average GD patients (e.g. severe Grave's orbitopathy, large & compressive goiters) | withdrawal from project, death, change of health insurance, move | | |
| N generally: N SDM-Q-9 &/-Doc relevant: | N = 93 (46 usual care, 47 intervention), after exclusion: N = 68, (37 usual care, 31 intervention); TAU: T1 = 31 post-encounter surveys (26 video-recordings); IG: T1 = 23 post-encounter surveys, (28 video recordings); SDM-Q-9 & -Doc relevant:  TAU: 28, IG: 23 | T0: N = 6542 contacted, 2188 responded (33,4%); IG: T0 = 496, T1 = 340, T2 = 309; CG1: T0 = 781, T1 = 479, T2 = 408; CG2: T0 = 911, T1 = 566, T2 = 488 | | |
| % women | TAU: 78%, IG: 81% | IG: 59,1%, CG1: 59,3%, CG2: 58,7% | | |
| education | TAU: high school = 30%, College = 30%, 4 year College = 10%, Graduate = 30%; IG: high school = 22%, College = 40%, 4 year College = 15%, Graduate = 24% | IG: without = 8,7%, secondary sch. = 71,7%, high school = 14,5%, A-level= 5,3%; CG1: without = 10%, secondary sch. = 68,7%, high school = 16%, A-level = 5,3; CG2: without = 8,8%, secondary sch. = 71,6%, high school = 14,9%, A-level = 4,7% | | |
| age (mean, SD & range) | overall: 42.8 (± 15) (18-76); TAU: 41.8 (± 14) (18-72); IG: 44.1 (± 16) (19-76) | IG: 62,9 (± 15,7), CG1: 62,8 (± 15,4), CG2: 63,3 (± 15,2) | | |
| specific health-care-provider | endocrinologists | IG: clinician chosen by patient as "clinician of trust" (GP); CG1 & CG2 = GP | | |
| inclusion criteria | working in the Maya Clinic, expertise in management of thyriod disorders | IG: clinician taking part in the integrative care system; CG1 & CG2 = GP | | |
| exclusion criteria | not reported | not reported | | |
| N | 9 endocrinologists filled out SDM-Q-Doc: TAU: 30, IG: 32 | not reported | | |
| % women | not reported | not reported | | |
| age (mean, range & SD) | not reported | not reported | | |
| test-statistics used | fisher's exact test statistics for categorial and mean (0.95 CI), median (IQR) with wilcoxon rank sum for continuous outcomes | 2-factorial ANOVA with the repetition factors time (3 points of measurement) and group (3 groups), alpha = 0.05; non-responder analysis (ANOVA), analysis of sensitivity for missing data imputation, sensitivity to change analysis without imputation of missing data | | |
| SDM-Q-9&/-Doc test scores (transformed score; mean, SD) | IG mean: patients: 44 (42, 47)* & clinicians: 44 (40, 51)*; TAU mean: patients: 42 (38, 47)* & clinicians: 42 (38, 47)*; *transformed after extraction | IG: T0 = 72,4 (±24,2), T1 = 68,9 (±24,9), T2 = 65,8 (±28,1); CG1: T0 = 71,1 (±25,3), T1 = 68,7 (±24,5), T2 = 69,2 (±26,7); CG2: T0 = 69,0 (±25,1), T1 = 67,7 (±25,0), T2 = 66,1 (±28,3) | | |
| significances | no significant effect of intervention on SDM-Q-9 & -Doc (analysis of video-recordings showed greater patient involvement- OPTION Scale) | no significant effect for intervention; significant effect of time: experienced involvement decreased independently of group (intervention) over time (no significant interaction-effect) | | |
| effect sizes | Mean difference IG-TAU: patients: 0.99 (CI 95%, -0.98, 3.0) p = 0.47, clinicians: 1.4 (CI 95%, -1.5, 4) p = 0.18,(OPTION Scale (patients): 5 (CI 95% 0.2-10) p = 0.02) | time-effect: p < 0,01 (significant)  group-effect: p = 0,31;interaction-effect: p = 0,17 (with high statistical power due to sample size, ƞ² = 0,01) | | |
| drop out | TAU: (N = 46) 9 patients did not have GD; (N = 37) 6 did not fulfill survey, (8 refused recording, 3 technical issues); IG: (N = 47) 14 did not have GD, 2 other exclusion criteria; (N = 31) 8 did not fulfill survey, (2 refused recording, 1 technical issue) | N = 6542 contacted, 2188 responded (33,4%) - 66,6% non-responders, probably due to acquisition by post without premonition; later drop-out: T1 36,6%; T2 41,1% --> low acceptance of instruments and no participation | | |
| knowledge about specific decisional topic (pre-post?) | the same in both groups, (only question about possible complications was answered slightly better in IG [p = .04]) | not applicable | | |
| interpretation of results | DA did not have an effect on self-reported SDM (SDM-Q-9 &-Doc), but on actual SDM in consultation (OPTION Scale) | no evidence for effects of "Gesundes Kinzigtal"-project on patient's perceived participation | | |
| reported study-limitations concerning SDM-Q-9 &/-Doc | many patients had a consultation about GD before the study - patients with  less knowledge could have derived greater benefit from DA, selection bias (no randomisation), small sample size (limited statistical power) | sensitivity to change: 1) instruments were built in controlled situations but data in this study was extracted in every-day situations; 2) variables of SDM are situational and influenced by the disease in question --> sensitivity to change analysis showed independency to procedure: decrease in SDM over time: 1) change of expectations about the consultations; high age of sample, 2) short SDM intervention, not enough to change behaviours of clinicians over time, 3) intervention aiming at patient activation rather than SDM, 4) higher age & lower education have been found to go with lower SDM interest | | |
| Rating via Quality Tools: | Poor | Fair | | |

| Study, year of publication: | Körner et. al 2012 (37) | Körner et. al 2014 (38) |
| --- | --- | --- |
| authors | Körner M., Ehrhardt H., Steger A.-K., Bengel J. | Körner M., Wirtz M., Michaelis M., Ehrhardt H., Steger A.-K., Zerpies E., Bengel J. |
| title | A multicentre-cluster randomized controlled study to evaluate a train-the-trainer programme for implementing internal and external participation in medical rehabilitation | Interprofessional SDM train-the-trainer program: "Fit for SDM": Provider satisfaction and impact on participation |
| study aim & research questions | evaluate the interprofessional SDM training programme "fit for SDM" in two steps: 1) train the trainers (&evaluate the intervention with feedback of trainees), 2) evaluate the impact of the intervention on internal (interprofessional SDM) & external (patient-professional SDM) participation | evaluate the train-the-trainer programme "Fit for SDM" for implementing internal (patient - HCP) & external (within treatment team) participation: evaluate the intervention effect on external and internal participation of patients and staff |
| country | Germany | Germany |
| primary/specialty care in-/out-patient care | specialty care in-patient care | specialty care in-patient care |
| medical condition/specific decisional context (treatments) | not reported | not reported |
| instrument version used (1=SDM-Q-9, 2= SDM-Q-Doc, 3= both) | 1 | 3 |
| adaptation of instrument (form) | modified for providers of SDM training  (implementation of external participation from the provider's perspective) | SDM-Q-9 & -Doc modified to assess SDM from the all HPC's perspectives |
| language/translation | German | German |
| former psychometrical testing | not reported | not reported |
| study type (e.g. control group, randomisation[level], stratification, blinding?) | Step 1: cross-sectional study; Step 2: multi-centre cluster-randomised controlled study (RCT) (randomisation at clinic-level) | multi-centre cluster-randomised controlled trial (RCT) (randomisation at clinic-level, patients blinded to IG/CG, matched pairs in clinics, no stratification) |
| points of measurement (SDM-Q-9 &/-Doc directly after treatment?) | T0 = pre-intervention, T1 = post-intervention --> exact time of measurement is not reported | T0 = pre-intervention, T1 = post-intervention, T2 = 6 months follow-up (patients only),--> questionnaires given directly after encounter by GPs, patients answered via post |
| usage of SDM-Q-9 &/-Doc as primary/secondary outcome | primary --> step 2: external participation (SDM) | primary |
| other outcomes assessed (1= primary, 2= secondary outcome) & which points of measurement? | Step 1: instruct future trainers 1) satisfaction with training content & with trainers (self-compiled) 1) SDM competences (self-compiled) 1) general training assessment & usefulness (self-compiled); Step 2: SDM transfer to treatment-team 1) internal participation (involvement of HCP within interprofessional teams in treatment planning) (6 item, self-compiled), further items self-compiled on: application, content, organization of & satisfaction with the training programme | 1) external participation: SDM-Q-9/-Doc (modified), 1) internal participation: self-compiled 6 item Internal Participation Scale (IPS) for patients & professionals |
| if both versions used: did they measure the same situation? | not applicable | not reported but probable |
| handling of missing data SDM-Q-9 &/-Doc (Intention-to-Treat principle? ITT) | not reported | questionnaires with more than 30% missing were excluded, imputation using expectation-maximization algorithm --> no ITT reported |
| treatment as usual (TAU) | clinics which received no training in SDM | consultation as usual, waiting control clinics (no training given until end of survey) |
| description of intervention (patient-&/practitioner-level, goal, form & duration) | training for SDM-trainers "fit for SDM" *module 1:* training for SDM after Bieber et al. ; external participation theory, creating phrases, role-playing; *module 2:* training to transfer SDM to the decision-making processes in the treatment team (participatory leadership) & prepare executive providers for their role as trainer; duration: not reported | interprofessional train-the-trainer programme "Fit for SDM" (online access to instrument in references) |
| Recruitment (for cluster-randomisation: independent recruiter?) | 15 in-patient medical rehabilitation clinics in Southwest Germany (of which 12 participated in both staff surveys) nothing further reported | 92 clinics selected from www.rehabilitationskliniken.de, of which 36 replied, 22 expressed interest, 14 declined participation; further 5 declined: N = 17 clinics, paired by type of rehabilitation & size for randomisation, study coordinators elected by clinics, which received questionnaires; 11 clinics after further drop-out: 8 somatic fields (orthopaedics, metabolism, neurology, cardiology, oncology) and 3 psychosomatic fields (mainly addiction) --> no independent recruitment reported |
| Size & power-calculation (ICC) | not reported | not reported, ICC smaller than 0.1 (aside from 6 months follow-up, ICC = 0.12), no size or power calculations done beforehand |
| inclusion criteria | not applicable | chronic disease, being treated in in-patient rehabilitation, ≥ 18 years, sufficient German language, signed informed consent |
| exclusion criteria | not applicable | cognitive impairments |
| N generally:N SDM-Q-9 &/-Doc relevant: | not applicable | N patient survey T0 = 1419 T0 = IG: 754 (9 clinics), CG: 665 (8 clinics), responded: IG: 332 (44%), CG: 329 (50%); N patient survey T1 = 1280, T1 = IG: 615 (6 clinics), CG: 665 (7 clinics), responded: IG: 224 (36%), CG: 299 (45%); N patient survey T2 = 1280 (new cohort), T2 = IG: 615 (5 clinics), CG: 665 (7 clinics), responded: IG: 199 (32%), CG: 342 (51%); after missing data analysis: IG: T0 = 158, T1 = 199, T2 = 168 CG: T0 = 244, T1 = 264, T2 = 293 |
| % women | not applicable | IG: T0 = 40,6%, T1 = 41,6%, T2 = 47,0%; CG: T0 = 33,1%, T1 = 33%, T2 = 38,6% |
| education | not applicable | IG: low --> T0 = 45,2%, T1 = 47,2%, T2 = 46,4%, middle --> T0 = 35,5%, T1 = 28,9%, T2 = 31,3%, high --> T0 = 19,4%, T1 = 23,9%, T2 = 22,3%, CG: low --> T0 44,6%, T1 = 46,4%, T2 = 52,1%, middle --> T0 = 34,3%, T1 = 28%, T2 = 30,5%, high --> T0 = 21,2%, T1 = 25,7%, T2 = 17,5% |
| age (mean, SD & range) | not applicable | IG: T0 = 57.1 (13.8), T1 = 58.7 (13), T2 = 61.5 (13.2), CG: T0 = 53.6 (12.7), T1 = 57.5 (13.7), T2 = 55.3 (13.9) |
| specific health-care-provider | step 2: physicians: 17,3% (31); nurses: 14% (25); psychosocial therapists: 27,9% (50); physical therapists 22,3% (40); others 18,4% (33) | physicians, nursing staff, physical therapists, sport teachers, masseurs, psychologists, other psychosocial therapists, dietitians, social workers |
| inclusion criteria | Working in rehabilitation clinics in Southwest Germany | working in the rehabilitation clinics with direct patient treatment |
| exclusion criteria | not reported | not reported |
| N | 12 clinics participating in both steps of intervention, N step 2 = 179 (questionnaires returned &complete), IG: 6 clinics, N = 69, CG: 6 clinics, N = 110 | N staff survey T0 = 662, T0 = IG: 355 (9 clinics), CG: 307 (6 clinics), responded: IG: 144 (41%), CG: 114 (37%) ; N staff survey T1 = 506, T1 = IG: 217 (6 clinics), CG: 289 (6 clinics), responded: IG: 73 (34%), CG: 116 (40%);Analysed after missing data analysis: IG: T0 = 82, T1 = 58, CG: T0 = 113, T1 = 110 |
| % women | step 2 in general: 64,8% (116); IG: 56,5% (39), CG: 70% (77) | IG: T0 = 52.4%, T1 = 58.6%; CG: T0 = 61,9%, T1 = 70% |
| age (mean, range & SD) | most between 36 & 55 years, with 5+ years in the clinic (mean, range and SD not reported, but age-table given) | most between 36 & 55 years, with more than half of them 5+ years in the clinic, (mean, range & SD not reported, but age table given) |
| test-statistics used | univariate analysis of variances: ANOVA (prae/post, IG/CG, occupational groups, age, gender, job tenure) | univariate analysis of variance (ANOVA), bivariate comparisons (chi-square-test, Phi, contingence coefficient CC) multivariate analysis of variances (MANOVA) to investigate differences in internal & external participation between IG & CG using partial eta square (ƞ²) further ANOVAs for occupational groups & clinics |
| SDM-Q-9&/-Doc test scores (transformed score; mean, SD) | T0 = IG: 63.7 (± 21.6), CG: 67.9 (± 21.1), T1 = IG: 75.2 (± 12.4) CG: 67.7 (± 22.5) | patient-survey: IG: T0 = 55.6 (26.2), T1 = 57 (26.4), T2 = 57.5 (26.4); CG: T0 = 59.1 (26.3), T1 = 59 (25.2), T2 = 58.3 (27.7)  staff-survey: IG: T0 = 62.5 (22), T1 = 72.9 (17.3); CG: T0 = 67.2 (21.6), T1 = 67.3 (22.5) |
| significances | no significant effect overall pre-post females rated external participation significantly higher after intervention than males, all occupational groups evaluated external participation after intervention better than before, with nurses significantly higher | small significant effect on external participation for staff after training (CG remained unchanged, significant interaction-effect), highest enhancement of external & internal participation through training for nurses, who began with mean of 43 external & 58 internal & showed 71 in both |
| effect sizes | 7.6 points higher (63.6 to 71.2) in IG after intervention compared to CG (F*period* x *group* (1) = 2.806, p = .095, ƞ² = .008), gender: mean diff. female: 16.6, male: -3.7, F*period* x *group* x *gender* (1) = 7.563, p = .006, ƞ² = .021,occupational groups: (F*occ. group* (4) = 8.372, p < .001, ƞ² = .089),nurses: F*period* x *group* (1)= 5.487, p = .023, ƞ² = .089 | staff: F*group* x *period: p = 0.028, ƞ² = .014;* nurses: F*group* x *period*: p = 0.022, ƞ² = .092 |
| drop out | questionnaires overall: 506, rate of return: 189 (37%),IG: 73 (34%), CG: 116 (40%), completion: 179, IG: 69, CG: 110 | patient survey questionnaires: IG: T0 = 66%, T1 = 64%, T2 = 68%; CG: T0 = 50%, T1 = 55%, T3 = 49%; staff survey questionnaires: IG: T0 = 59%, T1 = 66%; CG: T0 = 63%, T1 = 60% |
| knowledge about specific decisional topic (pre-post?) | not applicable | not applicable |
| interpretation of results | The training was evaluated positively; interprofessional training can be recommended for staff in rehabilitation clinics in order to enhance external participation in patient-provider interaction. | The training programme had a positive effect on external participation for HCP. Patient's results did not confirm this, but reported better patient-centred teamwork (internal participation) after staff had been trained. Nurses benefitted most, probably because of lower base rates and special appreciation of being given the opportunity to participate. Differing evaluation by staff of both participation forms confirms subcultures in clinical teams which hamper interprofessional education and practice. A change of training programmes is required to implement patient-centred treatment. |
| reported study-limitations concerning SDM-Q-9 &/-Doc | selection bias: survey was voluntary, only 12 clinics, possibly not reaching all team members. generalizability: small sample, low return rates (staff expressed high scepticism about anonymity, staff changes in clinics). statistical bias: CG is bigger than IG | low return rates (only 1/3 of clinics answered query so only 4 clusters could be built) resulting in a high difference in overall number of staff and patients (lower validity and generalizability), staff could not be matched due to anonymity of staff survey, staff expressed doubt about anonymity, voluntariness resulting in motivated clinics (selection bias), not all HCPs could be reached by trainings, economic difficulties and changes in staff teams |
| Rating via Quality Tools: | Poor | Poor |

| Study, year of publication: | Tinsel et. al 2012 |
| --- | --- |
| authors | Tinsel I., Buchholz A., Vach W., Siegel A., Dürk T., Buchholz A., Biebling W., Fischer K.-G. |
| titel | Shared decision making in antihypertensive therapy: a cluster randomised controlled trial |
| study aim & research questions | implement an evaluated SDM training programme for GPs in hypertension treatment 1) does this enhance patient's perceived participation?; 2) lower the blood pressure of patients? |
| country | Germany |
| primary/specialty care in-/out-patient care | treated but uncontrolled hypertension (24h-mean ≥ 130/80/ daytime-mean ≥ 135/85 /night-mean ≥ 120/70 mmHg) |
| medical condition/specific decisional context (treatments) | &/ relevant cardiovascular comorbidity (diabetes mellitus, coronary heart disease, heart attack, stroke/transient ischmic attack, peripheral arterial occlusive disease) |
| instrument version used (1=SDM-Q-9, 2= SDM-Q-Doc, 3= both) | 1 |
| adaptation of instrument (form) | not reported |
| language/translation | German |
| former psychometrical testing | not reported |
| study type (e.g. control group, randomisation[level], stratification, blinding?) | cluster randomised controlled trial (RCT) (randomisation at practice-level, no stratification, single-blinded: patients blinded to IG/CG) |
| points of measurement  (SDM-Q-9 &/-Doc directly after treatment?) | T0 = June-December 2009, pre-intervention, T1 = 6 months follow-up, T2 = 12 months follow-up, T3 = 18 months follow-up (last September 2011) --> questionnaires given directly after encounter by GPs, answering-mechanism not reported |
| usage of SDM-Q-9 &/-Doc as primary/secondary outcome | Primary |
| other outcomes assessed (1= primary, 2= secondary outcome) & which points of measurement? | 1) systolic Blood Pressure (24h-mean) T1-T3, 2) diastolic Blood Pressure (24h-mean) T1-T3, 2) patient's kowledge about hypptertension, T0-T3, 2) adherence (Medication Adherence Report Scale, MARS-D) T1-T3, 2) cardiovascular risk score (CVR) T1-T3, 2) other "self-reporting-instruments |
| if both versions used: did they measure the same situation? | not applicable |
| handling of missing data  SDM-Q-9 &/-Doc (Intention-to-Treat principle? ITT) | SDM-Q-9: imputation by at least 2 missing items; (mean score imputed); no systematic patterns of missing data found, ITT |
| treatment as usual (TAU) | no training in SDM, consultation as usual |
| description of intervention (patient-&/practitioner-level, goal, form & duration) | training for GPs: 6 hours, adapted to hypertensive treatment. Elements: 1) information on arterial hypertension, 2) physician-patient (risk) communication, 3) prcoess steps of SDM, 4) motivational interviewing, 5) introduction of decision table (to lower CVR), 6) role-plays simulating physician-patient consultation; further: flyer and recommendation for implementing a cardiovascular risk calculator which includes elements of SDM |
| Recruitment (for cluster-randomisation: independent recruiter?) | patients: via GPs. GPs: 115 by post - 68 responded; in South-West Germany --> no independent recruitment reported |
| Size & power-calculation (ICC) | N = 1200, 20% drop-out at practice & patient level each --> N = 788; effect sizes 0.3/0.35, power 74/87% (ICC 0,05) Bonferoni-Correction --> ICC of 0.05 in sample size alculation |
| inclusion criteria | repeated prescription of antihypertensive medication, ≥ 18 years, insured by a stationary health fund (exception Bundesknappschaft & See-Health-Insurance), understanding of the German language |
| exclusion criteria | dementia, mental handicap, short life-expectancy |
| N generally: N SDM-Q-9 &/-Doc relevant: | N patients = 1120 (552 intervention, 568 control), IG: T0 = 451, T1 = 363, T2 = 333, T3 = 301, CG: T0 = 489, T1 = 368, T2 = 295, T3 = 269 |
| % women | IG: 53,3%, CG: 55,3% |
| education | IG: low = 62,7%; middle = 24,1%; high = 13,2%, CG: low = 63,8%; middle = 22,6%; high = 13,6% |
| age (mean, SD & range) | IG: 63.8 (± 12.1), CG: 65.0 (± 12.4) |
| specific health-care-provider | General Practitioners (most associated with Division of General Practice of the University of Medical Centre Freiburg) |
| inclusion criteria | Southwest Germany, offering the full spectrum of a health care services, non-participation in another study implementing an SDM training as intervention |
| exclusion criteria | not reported |
| N | N = 37 (18 IG, 19 CG), IG: T0: 18, T1: 17, T2: 17, T3: 17, CG: T0: 19, T1: 19, T2: 18, T3: 18 |
| % women | not reported |
| age (mean, range & SD) | not reported |
| test-statistics used | mixed-model with repeated measurements, clustering effects considered, change from baseline, mean-effect over all follow-ups included: patient-data with baseline and at least one follow-up 1) sensitivity-analysis: endpoints T1 (BP,CVR,MARS-D) re-analysed using T0 data as baseline, 2) primary endpoints re-analysed for prognostical factors |
| SDM-Q-9&/-Doc test scores (transformed score; mean, SD) | IG: T0 = 73.00 (±17.66), T1 = 73.03 (±19,54); T2 = 70.51 (±20,98); T3 = 71.71 (±20.59),  CG: T0 = 70.67 (±20.24), T1 = 66.55 (±21.34), T2 = 67,20 (±20.00), T3 = 66.60 (±20.71) |
| significances | no significant effect for intervention on perceived participation |
| effect sizes | change from T0 3.11 points higher in intervention group, 97,5% CI, (-2,37; 8,61), p = 0.203 |
| drop out | 13 GPs and 529 patients before T0, 313 patients invalid baseline scores or controlled treated hypertension, 1 GP from intervention, 1 GP from control --> Drop-out rate around 15% (IG & CG), differential Drop-out < 15% |
| knowledge about specific decisional topic (pre-post?) | increased slightly in both groups: IG: +1.33 points, (95%,CI [-0,37; 1.72]) - no significance |
| interpretation of results | The SDM training had no effect on either patients' participation or blood pressure; effects found were rather due to a decrease in participation in the control group |
| reported study-limitations concerning SDM-Q-9 &/-Doc | GPs associated with Division of General Practice of the University of Medical Centre Freiburg are probably more open-minded and more open to improving their skills (external validity), no blinding of GPs possible, no objective measurement of GPs' change in behaviour according to SDM principles, training was probably too short to change behaviour of GPs |
| Rating via Quality Tools: | Poor |
