# Supplementary material for: Use of the 9-item Shared Decision Making Questionnaire (SDM-Q-9 and SDM-Q-Doc) in intervention studies—A systematic review
Source: PLoS One. 2017 Mar 30;12(3):e0173904. doi: 10.1371/journal.pone.0173904 (PMC5373562; doi:10.1371/journal.pone.0173904)
Supplement: S7 Table — (DOCX) [file pone.0173904.s008.docx]

**S7 Table. Data Extraction Sheet for Study Protocols.**

| Study Protocol, year of publication | den Ouden et. al 2015 | Drewelow et. al 2012 |
| --- | --- | --- |
| authors | den Ouden H., Vos R. C., Reidsma C., Rutten EHM G. | Drewelow E., Wollny A., Pentzek M., Immecke J., Lambrecht S., Wilm S., Schluckebier I., Löscher S., Wegschneider K., Altiner A. |
| title | Shared decision making in type2 diabetes with a support decision tool that takes into account clinical factors, the intensity of treatment and patient preferences: design of a cluster-randomized (OPTIMAL) trial | Improvement of primary health care of patients with poorly regulated diabetes mellitus type 2 using shared decision making - the DEBATE trial |
| study aim & research questions | to evaluate if OPTIMAL, a decision support tool which takes treatment intensity, patient's clinical characteristics & preferences into account does increase the proportion of met treatment targets in Type 2 Diabetes Mellitus patients. 1) effect of SDM on a multi-faceted DST on individualised treatment targets for HbA1c, blood pressure, LDL-cholesterol & identify determinants, 2) effect on treatment satisfaction, quality of life, health status, well-being, coping styles, 3) SDM knowledge of the GPs of both treatment groups after 24 months | the DEBATE trial: test if the intervention is able to 1) reduce HbA1c-value of the included patients by 0,5, 2) increase the participation level of the patients in SDM |
| country | Netherlands | Germany |
| primary/specialty care in-/out-patient care | primary care out-patient care | primary care out-patient care |
| medical condition/specific decisional context (treatments) | Type 2 Diabetes Mellitus (ADDITION intensified treatment or Dutch Guidelines treatment) | Type 2 Diabetes Mellitus(--> no specific treatment-options, overall compliance & health status) |
| instrument version used (1=SDM-Q-9, 2= SDM-Q-Doc, 3= both) | 3 (and observer usage via video-taping) | 1 |
| adaptation of instrument (form) | not reported | not reported |
| language/translation | Dutch | German |
| former psychometrical testing | not reported | not reported |
| study type (e.g. control group, randomisation[level], stratification, blinding?) | multi-centre cluster-randomised [controlled] trial [(RCT)] (randomisation at practice-level, no stratification, no blinding, 2 years follow-up) | multi-centre cluster-randomised controlled trial (RCT) (randomisation at practice-level, no stratification, only by gender if more than 15 patients per GP are eligible, no blinding) |
| points of measurement  (SDM-Q-9 &/-Doc directly after treatment?) | T0 = pre-intervention (IG: patients & GPs), T1 = 12 months follow-up (IG: GPs, SDM-Q-Doc),T2 = 24 months follow-up (IG & CG: patients), further: video-taping of one yearly-consultation --> exact time of measurement is not reported | T0 = pre-intervention, T1 = 6 months follow-up, T2 = 12 months follow-up, T3 = 18 months follow-up, T4 = 24 months follow-up--> data assessed via phone call after T0-T4, exact time of measurement is not reported |
| Use of SDM-Q as primary/secondary outcome | primary: patients (interaction of SDM), secondary: General Practitioners | secondary |
| other outcomes assessed (1 = primary, 2 = secondary) | 1) 3 treatment goals: HbA1c, blood pressure, total cholesterol, 2) 5 treatment goals: + body weight, smoking, 2) Diabetes Treatment Satisfaction Questionnaire (DTSQ), Audit of Diabetes Dependant Quality of Life (ADD QoL-19), Well-being-Questionnaire (W-BQ12), European Quality of Life (EQ-5D), Short Form-36 (health status), Diabetes Coping Measurement Questionnaire (DCMQ), 2) SDM ability of GPs: Level of SDM-knowledge/attitude (Control Preference Scale CPS; SDM-Q-9 & Doc), 2) observer based SDM rating of video-taped consultations by observer using SDM-Q-9 & socio demographic data | 1) HbA1c-level (T0-T4),2) EQ-5D (health status) & PAID (problem areas in diabetes) (T0, T2,T4), 2) PEF-FB-9 & PACIC-D (aspects of empowerment) (T0-T4), 2) BÄK Questionnaire (T0 & T4), 2) Pharmacotherapy (T0, T2, T4), 2) Cardiovascular Risk Prognosis (T0, T2, T4) & socio demographic data |
| if both versions used: did they measure the same situation? | yes | not applicable |
| handling of missing data (SDM-Q) (Intention-to-Treat principle? ITT) | not reported ITT | not reported ITT |
| treatment as usual (TAU) | treatment-as-before (according to Dutch guidelines or ADDITION intensive treatment algorithm from ADDITION study 2009) | GPs without training in SDM |
| description of intervention (patient-&/practitioner-level, goal, form & duration) | OPTIMAL - decision support tool:paper-based, for use of GP & patient, discuss treatment options & prioritising of treatment targets in 3 steps: 1) considering pros & cons & shared decision itself, 2) prioritising treatment targets to chosen option by individual preferences, lifestyle habits & values,3) treatment selection (detailed figure given); Training for GPs: 2 hours with role-play & DST | educative intervention: (following Elaboration Likelihood Model); aim: identify agenda & illness concept of patients with poorly regulated T2DM, change GPs communication patterns,Training for serving as a peer to a colleague, peer-visits, training to reflect SDM & treatment choices with patient, computer-based-decision aid (based on "arriba"), 2 group-training sessions after peer-visit |
| Recruitment (for cluster-randomisation: independent recruiter?) | 79 general practices participating in the ADDITION-europe study--> no independent recruitment reported | all GPs in area taking part in primary care (list by KV),study centres: Rostock, Düsseldorf, Witten; each study centre recruits 20 GPs from area (60 GPs in total with 13 patients per practice) --> no independent recruitment reported |
| inclusion criteria | 1. former ADDITION patient, T2DM 2002-2004, aged 50-70 years (receiving either intensified treatment or Dutch guidelines treatment), 2) patient aged 60-80 years 2012-2014, known T2DM for 8-12 years, not diagnosed in ADDITIONstudy | T2DM, HbA1c-level over 8.0, ability to give informed consent, sufficient German language skills |
| exclusion criteria | alcoholism, drug abuse, psychosis, personality disorder, other emotional/ psychological/intellectual problem which is likely to invalidate informed  consent/limits ability to comply, limited life-expectancy | severe co-morbidity with a life expectancy of less then 24 months |
| N & power-calculation (ICC) | N = 73 per group, p = 80%, alpha 0.05, CI = 95% --> within-clustercorrelation coefficient [1 (m-1)r] r = 0.025 (based on cluster correlation found in former ADDITION study) | 60 GPs (Drop-Out expected 10% = 54 GPs) with 13 patients each (Drop-Out expected 20% = 10 eligible patients). N = 780 patients (derived factor 1.9, ICC 0.1, average cluster size of 10; p = 80%) --> with ICC of 0.1 |
| specific health-care-provider | General Practitioners (GP) | General Practitioners (GP) |
| inclusion criteria | participated in the ADDITION-europe study and included at least 2/ more comparable patients from patients inclusion criteria 1) & 2) | specialist for general/internal medicine, practicing with GP KV-admission |
| exclusion criteria | not reported | not reported |
| N & power-calculation | not reported | 60 GPs needed (20 per study centre) |
| planned statistical analysis of SDM-Q-9 &/-Doc | differences in the intervention group: paired t-tests, differences between the groups: mixed models, interaction of SDM with: age, gender, education, duration of diabetes, comorbidities | secondary endpoints will be analysed like the primary endpoints: mixed model with repeated measurements, coefficient test (Maximum-Likelihood & missing-at-random-assumption),sensitivity-analysis (e.g. last-observation-carried-forward),analysing groups scatter plots to correct for cluster-effects |
| level of significance/relevance regarding SDM-Q-9&/-Doc results | p-value of <0.05 in t-test | not reported |
| Rating via Quality Tools: | Fair | Fair |

| Study Protocol, year of publication | Geiger et. al, 2011 | Goss et. al, 2015 |
| --- | --- | --- |
| authors | Geiger F., Liethmann K., Paschedag J., Kasper J. | Goss C., C., Ghilardi A., Deledda G., Buizza C., Bottacini A., Del Piccolo L.,  Rimondini M., Chiodera F., Mazzi M. A., Balalarin M., Bighelli I., Strepparava M. G., Molino A., Riorio E., Nortilli R., Caliolo C., Zuliani S., Auriemma A., Maspero F., Simoncini E. L., Ragni F., Brown R., Zimmermann C. |
| title | Investigating a training supporting shared decision making  (IT's SDM 2011): study protocol for a randomized controlled trial | INvolvement of breast CAncer patients during oncological consultations: a multicentre randomised controlled trial - the INCA study protocol |
| study aim & research questions | evaluate IT'S SDM 2011 intervention: 1) to enhance physicians communication behaviour & improve SDM, 2) validate determinant for SDM degree (SDM-Q-9),3) evaluate SDM regarding its effects on patients' decisional conflict & internal process of elaboration (Sub goals: validate short version of Uncertainty Profile questionnaire (UP24), yield data on interrelatedness of different perspectives on communication) | 1) evaluate pre-consultation intervention to increase involvement of breast cancer patients during consultation, 2) explore role of attending companions in the information exchange during consultation |
| country | Germany | Italy |
| primary/specialty care in-/out-patient care | specialty care out-patient care | specialty care out-patient care |
| medical condition/specific decisional context (treatments) | no specific condition/decisional context/treatments reported | breast cancer, physician expects decision in upcoming consultation (no specific treatment reported) |
| instrument version used (1=SDM-Q-9, 2= SDM-Q-Doc, 3= both) | 1 | 1 (and adapted version for companion) |
| adaptation of instrument (form) | not reported | version for companion of patient |
| language/translation | German | Italian |
| former psychometrical testing | not reported | not reported |
| study type (e.g. control group, randomisation[level], stratification, blinding?) | multi-centre [cluster]randomised controlled trial (RCT) (randomisation at physician-level, patients randomised in clusters via physicians, double-blinded, no stratification) | multi-centre randomised controlled trial (RCT) (randomisation at patient-level, stratified by centre,double-blinded) |
| points of measurement (SDM-Q-9 &/-Doc directly after treatment?) | T0 = pre-intervention, T1 = IG: intermediate assessment, CG: waiting assessment, T2 = IG: post-intervention, CG: intermediate assessment, T3 = IG: follow-up (6 months), CG: post-intervention, patients: partake only at one consultation, physicians: four consultations each--> exact time of measurement is not reported | T0 = pre-intervention, T1 = directly after consultation (SDM-Q-9 only T1), further: audio-recordings of consultations --> questionnaires given and gathered directly after encounter by assistant |
| usage of SDM-Q-9 &/-Doc as primary/secondary outcome | Secondary (patients, only in centres where UP24 is not applicable) | secondary |
| other outcomes assessed (1 = primary, 2 = secondary) | 1) MAPPIN'SDM inventory (objective judgement based on video-tapes,  mutual involvement in SDM physician & patient), 2) Decisional Conflict Scale (DCS), 2) UP24, patient's cognitive representation of decision related uncertainty) & socio demographic data | 1) number of questions asked on QPS topics, 2) number of unmet information needs, 2) Ability to cope with illness (PEI), 2) patient involvement (SDM-Q-9 & OPTION Scale), 2) satisfaction with decisions made (SWD), 2) Recalling & understanding of information (Recall Questionnaire), 2) whether the patient asked their selected questions (self-composed 3 items), 2) perceived patient-doctor relationship (PDRQ-9) & Difficult Doctor Patient Relationship Questionnaire (DDPRQ-10), 2) oncologists answered questions about perceived anxiety, depression, emotional distress of patients & difficulty in answering patient's questions (self-composed), 2) perceived role preference of patients (CPS)2) duration of consultation & socio demographic & clinical data |
| if both versions used: did they measure the same situation? | not applicable | not applicable |
| handling of missing data (SDM-Q) (Intention-to-Treat principle? ITT) | not reported | not reported ITT |
| treatment as usual (TAU) | consultation as usual, waiting control group | control sheet: "please indicate the issues which you want to discuss today with your oncologist"; oncologists perform treatment as usual |
| description of intervention (patient-&/practitioner-level, goal, form & duration) | "doktor-mitSDM" training curriculum: built on evidence-based patient information (EBPI) & SDM, didactics from psychotherapy education techniques, aims at improving physicians communication behaviour via feedback1) manual: background information, skills, examples, 2) training video: examples evaluated by MAPPIN'SDM framework, 3) face to face feedback: 15 minutes, on taped consultation, concrete feedback in 6 steps | question prompt sheet (QPS):list of 50 specific questions, divided by topics (diagnosis, treatment, contribution of patient & lifestyle, prognosis, other issues), patient & companion are invited to select & encircle the salient questions |
| Recruitment (for cluster-randomisation: independent recruiter?) | 7 university outpatient clinics in Germany, (oncology, gynecology, psychiatry, neurology, dentistry, radiology)--> no independent recruitment reported | Oncology Out-Patient Services, 3 oncology departments in Northern Italy (in Veneto & Lombardia) with 2-5 oncologists, (started June 2011, end 2 years/sample limit reached) |
| inclusion criteria | physician expects a medical decision to be negotiated within the encounter | all female patients with breast cancer at their first consultation attending oncology out-patient clinics, have undergone breast surgery, aged ≥ 18 to 75 years |
| exclusion criteria | not reported | age ≥ 76; presence of metastasis or relapse; severe mental detoriaton; comprehension difficulties of the Italian language |
| N & power-calculation (ICC) | for study question 2 (SDM-Q-9): N = 76 (alpha: 0.05, p = .85) --> no calculation of cluster-effects (ICC) reported | N = 300 (15% Drop-Out), 250-260 patients to complete the study, with 130 patients per group 30 patients, 10 per centre, to assess the number of questions asked beforehand, increase of 30% in questions asked as goal (p = 80%, alpha: 0.05) |
| specific health-care-provider | oncologists, gynaecologists, psychiatrists, neurologists, dentists, radiologists | oncologists |
| inclusion criteria | not reported | not reported |
| exclusion criteria | not reported | not reported |
| N & power-calculation | for study question 2(SDM-Q-9): N = 36, (alpha: 0.05, p = .85) | not reported |
| planned statistical analysis of SDM-Q-9 &/-Doc | training effects pre-post intervention: Student's t-test (grouped scatter plots for cluster analysis), no further information on analysis of SDM-Q-9 reported | second endpoints: multi-level analyses to assess the specific effect of individual oncologists. no further information on analysis of SDM-Q-9 reported |
| level of significance/relevance  regarding SDM-Q-9&/-Doc results | not reported | not reported |
| Rating via Quality Tools: | Fair | Good |

| Study Protocol, year of publication | Löffler et. al 2014 | Savelberg et. al 2015 |
| --- | --- | --- |
| authors | Löffler C., Drewelow E., Paschka S. D., Frankenstein M., Eger J., Jatsch L., Reisinger E. C., Hallauer J. F., Drewelow B., Heidorn K., Schröder H., Wollny A., Kundt G., Schmidt C., Altiner A. | Savelberg W., Moser A., Smidt M., Boersma L., Haekens C., van der Weijden T. |
| title | Optimizing polypharmacy among elderly hospital patients with chronic disease- study protocol of the cluster randomized controlled POLITE RCT | Protocol for a pre-implementation and post-implementation study on shared decision making in the surgical treatment of women with early-stage breast cancer |
| study aim & research questions | evaluate effectiveness of the POLITE intervention: to reduce the number of long-term drugs among multimorbid & chronically ill patients aged 65+ years | pilot-test & optimise strategies for implementing SDM for patients with  early-stage breast cancer in a clinical setting: 1) what are the perceived barriers, facilitators, needs, preferences of patients & professionals (integration of DA, model of SDM integration, which coachings/instructions for SDM for professionals are needed),2) impact of implementation of DA on SDM process, patients' knowledge & decisional conflict, 3) extent to which DA produces changes in intended & final treatment decision by doctors & patients |
| country | Germany | Netherlands |
| primary/specialty care in-/out-patient care | primary & secondary care, in-patient care | specialty care, (primary therapy) in-patient care |
| medical condition/specific decisional context (treatments) | chronic diseases, multimorbidity, no specific condition, context: polypharmacy (long-term drugs) | surgical treatment of early stage breast cancer - mastectomy (breast removal) or lumpectomy (breast-conserving)with radiation |
| instrument version used (1=SDM-Q-9, 2= SDM-Q-Doc, 3= both) | 1 | 3 (although SDM-Q-Doc is not mentioned in name, but focus will be on the dyadic approach measurement of perceptions of patients and clinicians is reported) |
| adaptation of instrument (form) | not reported | not reported |
| language/ translation | German | Dutch |
| former psychometrical testing | not reported | not reported |
| study type (e.g. control group, randomisation[level], stratification, blinding?) | cluster-randomised controlled trial (RCT) (randomisation at ward-level, stratification, no blinding, [aside from T2 & T3 pharmacists], CTCN - Clinical Trial Centre North, University-Hospital Hamburg-Eppendorf will overlook proceedings) | historically controlled pre-/post-implementation study (preliminary survey for a RCT-Pilot study) (no randomisation, no stratification) |
| points of measurement (SDM-Q-9 &/-Doc directly after treatment?) | T0 = pre-intervention, (admission to hospital),T1 = discharge from hospital, T2 = 6 months follow-up,T3 = 12 months follow-up --> T0 data collection directly at admission, T2 & T3 data via phone call (SDM-Q-9) | T0 = pre-implementation, T1 = implementation, T2 = post-implementation --> exact time of measurement is not reported |
| usage of SDM-Q-9 &/-Doc as primary/secondary outcome | secondary | primary |
| other outcomes assessed (1 = primary, 2 = secondary) | 1) health related quality of life (EQ-5D) (T0, T2, T2), 1) difference in number of prescribed long-term pharmaceutical agents between IG & CG (T0-T4), 2) appropriateness of prescribed medication (PRISCUS list), Beers Criteria, MAI [T0-T4], patient satisfaction TSQM [T0, T2, T3], patient empowerment PEF-FB-9 [T0,T2,T3], patient autonomy IADL [T0, T2, T3], falls- frequency & severity [T0-T4], re-hospitalization [T2, T3], death [T1, T2, T3], & socio demographic data [T0, T2, T3] | 1) patient's knowledge about breast cancer (breast cancer information test) (T1), 1) Decisional Concflict Scale (T0, T1), 1) perceptions of patients & clinicians SDM (SDM-Q-9/Doc) (T0, T1), 1) process of SDM from independent observer from audiorecordings (Observer OPTION Scale) (T0, T1), 1) audit on actual decision taken, 1) time patients were reading the DA, 1) time professionals spent on consultation via audiorecordings, 1) feasibility of DA (patients) (T2),1) feasibility of DA (clinicians) focus-groups & face-to-face discussions (T2) |
| if both versions used: did they measure the same situation? | not applicable | yes |
| handling of missing data (SDM-Q) (Intention-to-Treat principle? ITT) | missing values will not be replaced ITT | not reported |
| treatment as usual (TAU) | patients will not receive a medication review, consultation as usual | consultation as usual, before implementation of SDM |
| description of intervention (patient-&/practitioner-level, goal, form & duration) | narrative-based medication review: (pilot tested) 30-45 minutes detecting potentially inadequate medication & identifying patients' preferences. The pharmacist prepares list of potential drugs to be stopped with help of a clinical decision support system which will be discussed with GP & hospital physician in charge of ward | instruments & activities to include SDM, Patient Decision Aid (by Maastricht University Medical Centre & Amsterdam Academic Medical Centre): easy-to-use website (link & password); comprehensive information: mastectomy & lumpectomy with radio therapy, pictures and graphics, verbal information, summarizing factsheet, option grid & questions to identify individual values,Training for professionals: instructions with e-learning component & integration suggestions of DA |
| Recruitment (for cluster-randomisation: independent recruiter?) | 2 major secondary HCPs in Mecklenburg-Western Pomerania, 4 hospitals , 30 patients per week per hospital consecutively at admission (started August 2014, 6 months baseline recruitment) --> yes, pharmacists via CTCN | 4 Dutch hospitals (western, central, southern areas) (pre-implementation = 3 months - N = 40, implementation = 5 months - N = 40; each hospital N = 10), patients will be identified at multidisciplinary meetings of breast cancer teams |
| inclusion criteria | ward of the participating centres, chronic disease & multimorbidity, ≥ 65 years, taking 5/more long-term drugs that are systematically acting, likely to spend 5 days in one of the participating hospitals | newly diagnosed breast cancer (stage 1/2) eligible for breast-conserving therapy or mastectomy as primary therapy, fluent in Dutch language |
| exclusion criteria | unable to take medication by themselves, not able to give legal informed consent, severe language difficulties, deafness, taking part in another clinical trial, with disease making polypharmacy unavoidable (active malignoma, acquired immunodeficiencies (HIV), hemodialysis, post-transplant patients, life expectancy ≤ 12 months) | not reported |
| N & power-calculation (ICC) | N = 1626 with drop out 5%: N = 1544 patients (p = 80%, alpha: 0.05), IG: 772 patients & CG: 772 patients (with further drop out 5%), in 42 wards (entity with stable medical personnel):IG: 21 wards & CG: 21 wards --> with ICC 0.1 | pre-implementation: N= 10,Implementation: N = 10, post-implementation: N = 4 per hospital from implementation-sample (N = 16) for qualitative measurements |
| specific health-care-provider | pharmacists (General Practitioners, hospital physicians in charge) | breast surgeons, radiation oncologists, nurse practitioners, nurses |
| inclusion criteria | not reported | taking part in the decision making process |
| exclusion criteria | not reported | not reported |
| N & power-calculation | only in form of wards, see above | not reported |
| planned statistical analysis of SDM-Q-9 &/-Doc | secondary endpoints will be analysed in an explorative way | not reported |
| level of significance/relevance  regarding SDM-Q-9&/-Doc results | not reported | not reported |
| Rating via Quality Tools: | Fair | Fair |
